# Totally implanted chemotherapy port catheters: literature review and report of four cases

**DOI:** 10.1093/jscr/rjab194

**Published:** 2021-05-10

**Authors:** Pezhman Farshidmehr, Zahra Omrani, Roozbeh Cheraghali

**Affiliations:** 1 Tehran University of Medical Sciences, Sina Hospital, Tehran, Iran; 2 Department of Surgery, Iran University of Medical Sciences, Tehran, Iran; 3 Golestan University of Medical Sciences, 5 Azar Hospital, Gorgan, Iran

## Abstract

Totally implantable catheters tend to be the most popular choice because once installed they allow permanent access to a deep vein, which is gained by puncturing the port rather than a vein.

In this article, we explain four cases of chemotherapy port complications: superior vena cava (SVC) syndrome in a metastatic colorectal cancer patient who presented with bilateral mastitis, snare technique for removal of migrated catheter line, carotid artery placement of a port in a 5-year-old child that was referred to our hospital from a pediatric center and adhesive port tip in the heart that finally we left the port *in situ*.

In SVC syndrome, treatment should be guided by the severity of symptoms, etiology of the obstruction, prognosis of the patient and treatment goals. We propose timely removal of port-a-cath following completion of intended chemotherapeutic regimen.

## BACKGROUND

Totally implantable catheters of the port-a-cath type tend to be the foremost popular choice because once inserted they permit permanent access to a deep vein, which is gained by puncturing the port rather than a vein [1]. Several complications associated with chemo port implantations include venous thrombosis, infection, catheter extravasation and dislodgement. [2] Superior vena cava (SVC) syndrome occurs when there is direct compression or obstruction of the SVC. It is usually suspected based on medical history and physical examination and can be confirmed by cross-sectional imaging. [3] The increased use of indwelling lines and pacemakers in recent years has also yielded more cases of SVC syndrome overall [4]. Removal of the port-a-cath is generally a short, uncomplicated procedure and should be done soon after completion of the intended regimen [5]. The incidence of port catheter dislodgement with subsequent migration to the heart is low with an estimated rate of up to 4.1% [6]. In many patients, it’s detected incidentally when patients undergo routine chest radiographs. The treatment of choice for port dislodgement is immediate retrieval of the distal migrated part, and percutaneous transvenous retrieval is considered the standard method because it’s generally easy, safe and least invasive with a high success rate [7]. In this article, we report four cases of port complications referred to us as vascular surgeons. Consent form was obtained from all patients.

## CASE PRESENTATION

**Figure 1:**
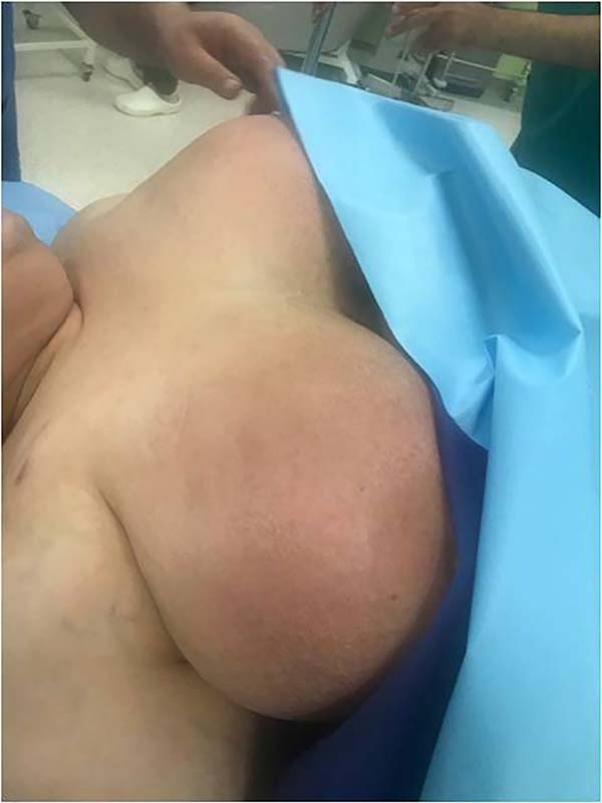
Bilateral mastitis, the chief complaint of the patient.

Case 1—right jugular central venous (CV) port was created for a 67-year-old woman due to metastatic colorectal cancer. She underwent a chemotherapy regimen for 8 weeks. Bilateral mastitis appeared after the end of the chemotherapy period. She also had a headache, sore throat, dyspnea and chest congestion. Upon arrival, the patient complained of shortness of breath, hoarseness and neck swelling. On physical examination, she was afebrile with a blood pressure of 139/86, heart rate of 90/min, respiratory rate of 23/min and oxygen saturation of 96% on room air. There was swelling in her head and neck, a plethora of her face, neck and shoulders. There were no carotid bruits, jugular venous distension, stridor, drooling, trismus or tracheal deviation (Fig. 1).

**Figure 2:**
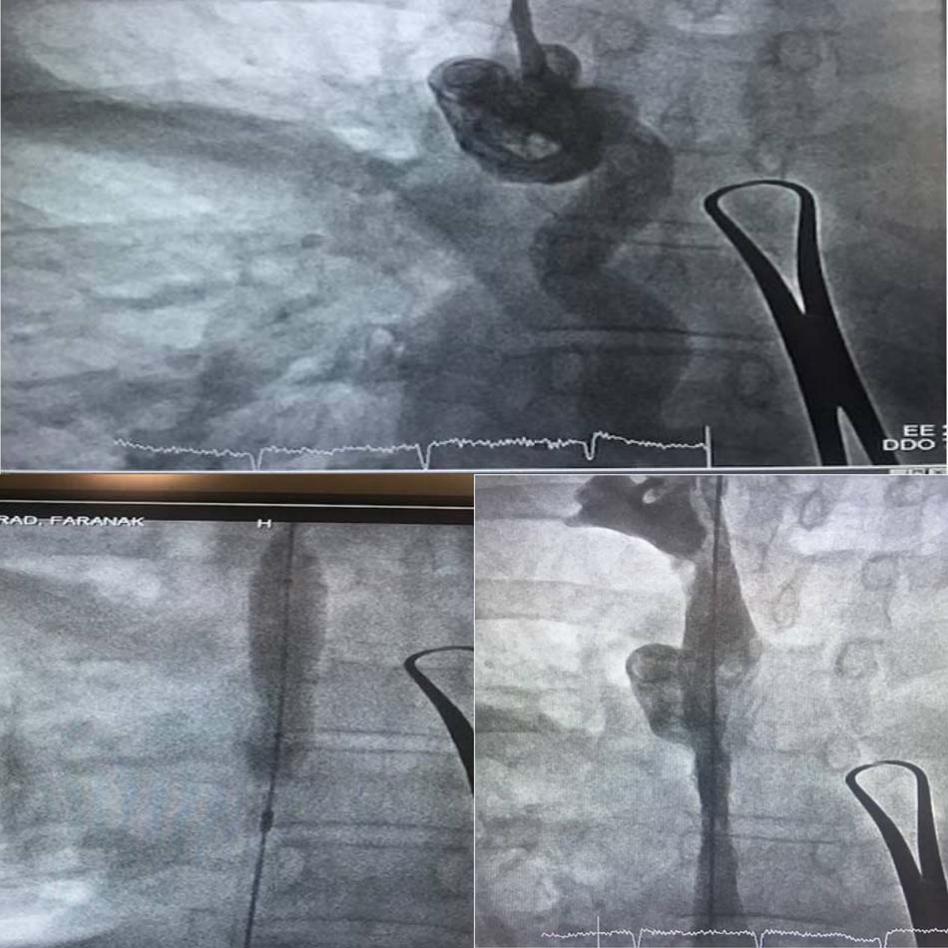
Balloon venoplasty to open SVC stenosis.

Magnetic resonance imaging was scheduled to evaluate brain metastasis. The venogram showed occlusion in the SVC. As the patient’s symptoms presented in <1 month, we passed a thrombolytic catheter through the port line, and the port was removed. Thrombolytic agent and heparin infusion were prescribed for 48 h. Repeated venogram revealed remained occlusion in SVC, so balloon venoplasty was done a day later (Fig. 2). The procedure was uncomplicated. The patient’s symptoms were remarkably improved at the time of discharge.

Case 2—a 60-year-old woman with breast cancer underwent a left mastectomy and an implantable chemotherapy port was inserted via right subclavian vein puncture, with fixation of the reservoir in the right thorax. She used her port for 12 months and was referred by an oncologist to explant the port catheter. The general surgeon missed the catheter when he wanted to separate the reservoir from the catheter, and vascular surgery consult was done. Chest radiograph was performed due to suspicion of catheter migration secondary to manipulated breakage of chemo port catheter. It showed that chemo port catheter had migrated from its original location to the right ventricle, traveling across the right atrium. The symptoms related to catheter migration that include palpitations, dyspnea, cough and chest discomfort were absent in this patient, and there was no electrocardiographic changes.

**Figure 3:**
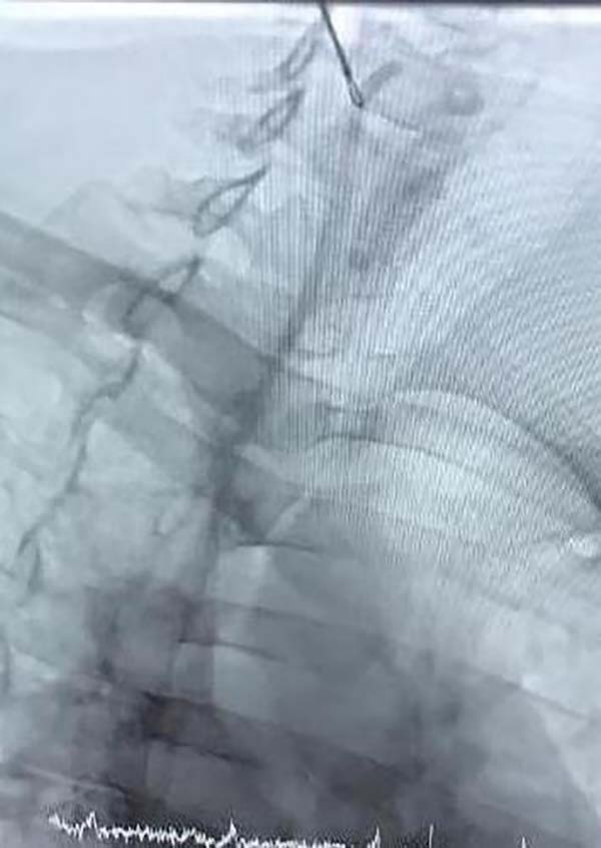
Snare-technique retrieval of chemo port catheter.

The right jugular vein was punctured, and a 6-Fr sheath was inserted. We removed the catheter from the right jugular vein. No major complication occurred during and after the procedure, and therefore, the patient was discharged on subsequent day (Fig. 3).

**Figure 4:**
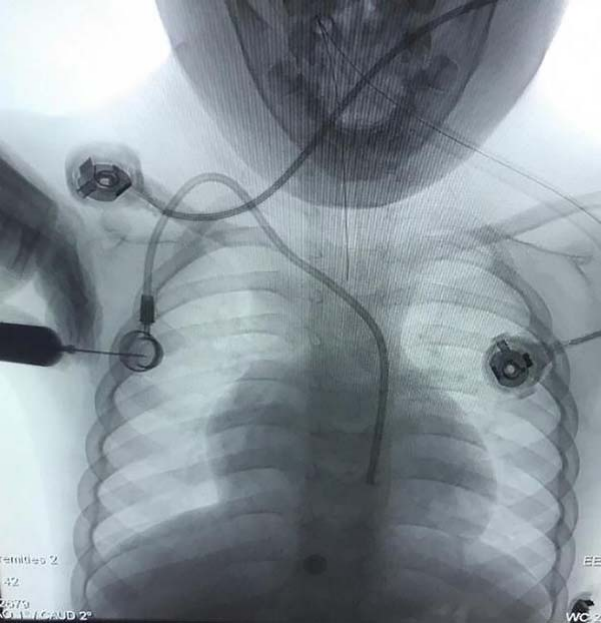
Chemotherapy port catheter in the carotid artery of a 5-year old child.

Case 3—a 5-year-old male child was diagnosed as having Hodgkin’s lymphoma during the evaluation of cervical lymphadenopathy and weight loss. He was planned for chemotherapy, and a central vein chemo port was implanted in a pediatric hospital. They took a chest X-ray (Fig. 4) after the procedure and understood the catheter is in the carotid artery instead of the jugular vein so they consulted with us and sent the child in <4 h to our vascular surgery operating room. We prepped and draped the neck of the child and removed the catheter from the carotid artery and packed it for 20 min. The child discharged the next day without any complication, and 10 days later in the clinic, he was fine.

Case 4—a 67-year-old woman was referred to us to remove her chemo port after 11 years. During colonoscopy for colon cancer screening, a small ulcerative sessile polyp was found in the right colon, and after surgery, the patient was in Stage 3 and undergone a period of a chemotherapy regimen. The patient missed the follow-up period, and her chemo port was left in her right chest. Under monitored anesthesia care and local anesthesia, an incision was made over the port site, ~2 cm inferior to the prior incision site. The port and catheter were identified covered with the chronic capsule. Subcapsular dissection allowed for complete freeing of the port, but significant resistance was felt upon pulling the catheter. As we first check the line of the catheter after freeing it before explantation of the reservoir, we found that the line tip is fixed. So we didn’t remove the port and left the port in place with its reservoir.

## DISCUSSION

SVC syndrome is diagnosed clinically with an in depth history and physical exam. However, imaging is required for a definitive diagnosis. Contrast venography can confirm the diagnosis of SVC syndrome and also for direct treatment and monitor the progression of therapy [4]. Computed tomography angiography or magnetic resonance angiography are superb modalities for diagnosis and may quickly be used. Our patient had typical history and physical exam, so we used ultrasound initially because it is quick, inexpensive and venography was done to verify the diagnosis and treating SVC thrombosis with endovascular balloon venoplasty. She had bilateral mastitis, she was referred to us later. As we weren’t certain about the accurate time of thrombosis, thrombolytic therapy was conducted first, but stenosis didn’t differ significantly in repeated venography. Hence, we decided to perform balloon venoplasty.

To date, the complication rate for the dislodged catheter of a chemo port has remained low with a prevalence of 0.4–4% [7]. The mechanism of catheter dislodgement and migration is not clear. Regardless of how, when a dislodged fragment is found on chest X-ray, early removal as soon as possible is important due to two reasons, one is to stop its distal embolization, which makes retrieval harder, and one more reason is that foreign bodies can cause septicemia, lung abscess, multiple pulmonary emboli, arrhythmias, cardiac wall necrosis resulting in perforation and sudden cardiac death [8]. Percutaneous endovascular retrieval has become a typical technique for foreign body removal. Now it’s a preferred method with a high success rate of 71–100% [2]. In our case, we used the snare technique for retrieval of the migrated catheter without vascular or cardiac complication. So every catheterization lab should have enough expertise to retrieve the migrated catheters albeit the patient is asymptomatic.

Arterial puncture or cannulation with a sheath introducer or CV catheter is related to potentially devastating consequences. This happens at the carotid or subclavian artery in ~0.1% to a quarter of cases: 30% of those patients are often expected to become symptomatic, and if so, the death rate reaches 20–40% [1]. One must check whether the punctured site is compressible (by ultrasound) or not before he removes the inserted device. Puncture of the subclavian artery during subclavian vein catheterization attempts occurs in 0.5–4% of the patients. The subclavian artery cannot be compressed; so, the subclavian approach should be avoided in anticoagulated patients. The location of a CV catheter with the Seldinger technique could also be a choice for prevention. Percutaneous closure, balloon occlusion, covered stent placement, thrombin injection or surgical repair are used for treatment [1]. Within the case described here, the kid was referred to us in <4 h so we didn’t perform the other diagnostic imaging as we’d lose the time, then the catheter was removed, and therefore, the site of catheter insertion was packed. If the catheter remained for quite 6 h, we required proximal control of the artery, before resecting the catheter line and repair its puncture site with open surgery or by endovascular techniques (balloon occlusion, covered stent placement).

Documented risk factors for the event of intravascular adhesion of a port-a-cath include younger age of insertion, increased port dwell time (>20 months), a diagnosis of acute leukemia and polyurethane catheter use [9]. Complications of forceful traction in attempted removal include vascular injury at the location of adhesion and catheter fragmentation, which can be avoided with the utilization of a guide wire [5]. During this case, we used a guide wire, but it neither passes the catheter line completely nor decreases in resistance of the catheter, so we decided to leave it in place. A single-center study of totally implantable venous access device removal by Wilson *et al*. [9] revealed no complications with retained catheters in place without venous thromboembolism prophylaxis at 6-year follow-up. Longer-term analysis has not been reported, and potential complications like local infection and venous thromboembolism cannot be definitively precluded.

## CONCLUSION

In SVC syndrome, treatment should be guided by the severity of symptoms, etiology of the obstruction, prognosis of the patient and treatment goals. We propose timely removal of port-a-cath following completion of intended chemotherapeutic regimen. In cases of indwelling catheters for extended periods (>20 months), appropriate preparation should be made in anticipating this complication (adherent) with removal, forewarning the anesthesiologist and ensuring endovascular and imaging equipment. It’s highly recommended to all surgeons and vascular surgeons to use ultrasound while puncturing the vein to reduce arterial puncture and multiple trials, which will cause pneumothorax, hematoma, increase risk of infection, neural injury, etc.

## ETHICS APPROVAL AND CONSENT TO PARTICIPATE

This case series was approved by the Tehran University of Medical Sciences research and Ethics Committee (TUMS Ethics Committee).

## CONSENT FOR PUBLICATION

Written informed consent was obtained from the patient for publication of this case report and any accompanying images. A copy of the written consent is available for review by the Editor-in-Chief of this journal.

## AVAILABILITY OF SUPPORTING DATA

Due to confidentiality agreements, supporting data can only be made available to researchers subject to a nondisclosure agreement. Details of the data and how to request access are available from Dr Roozbeh Cheraghali (roozbehcheraghali81@gmail.com) at Sina Hospital Cath Lab, Tehran, Iran.

## CONFLICT OF INTEREST STATEMENT

None declared.
